# Supplementary material for: In vivo single-cell CRISPR uncovers distinct TNF programmes in tumour evolution
Source: Nature. 2024 Jul 17;632(8024):419–28. doi: 10.1038/s41586-024-07663-y (PMC11306103; doi:10.1038/s41586-024-07663-y)
Supplement: Supplementary file 1 — The file contains Supplementary Figs. 1–11. [file 41586_2024_7663_MOESM1_ESM.pdf]

nature portfolio

https://doi.org/10.1038/s41586-024-07663-y

Supplementary information

In vivo single-cell CRISPR uncovers distinct TNF programmes in tumour evolution

In the format provided by the
authors and unedited

Supplementary Figure 1

a **Wholemount immunofluorescence analysis of mCherry clone distribution in the skin.**

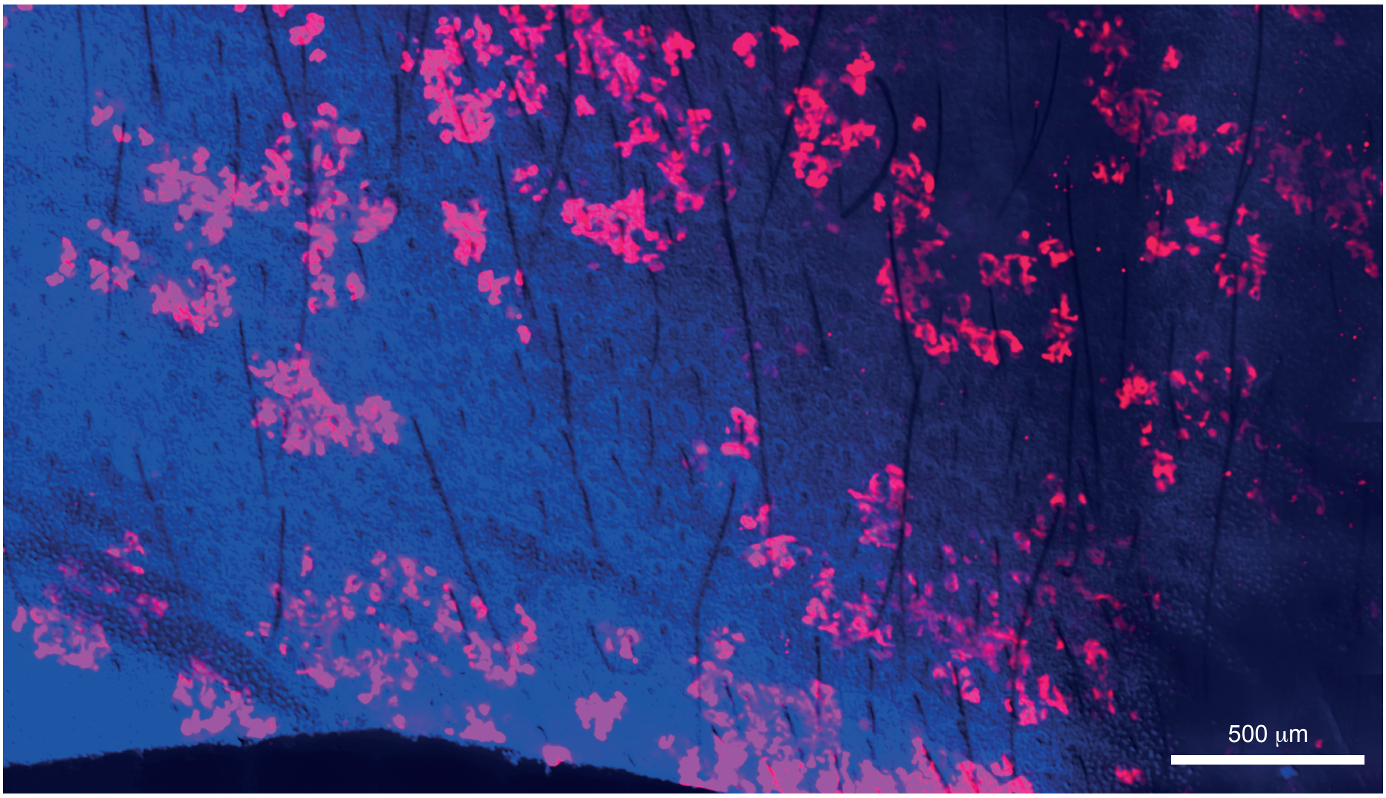

Supplementary Figure 1. Wholemount immunofluorescence of larger skin region.
a, Wholemount immunofluorescence staining of mCherry-positive clones in P4 skin, injected with the library of 500 sgRNAs. Immunofluorescence images provide a planar view across the epidermis.

Supplementary Figure 2

a

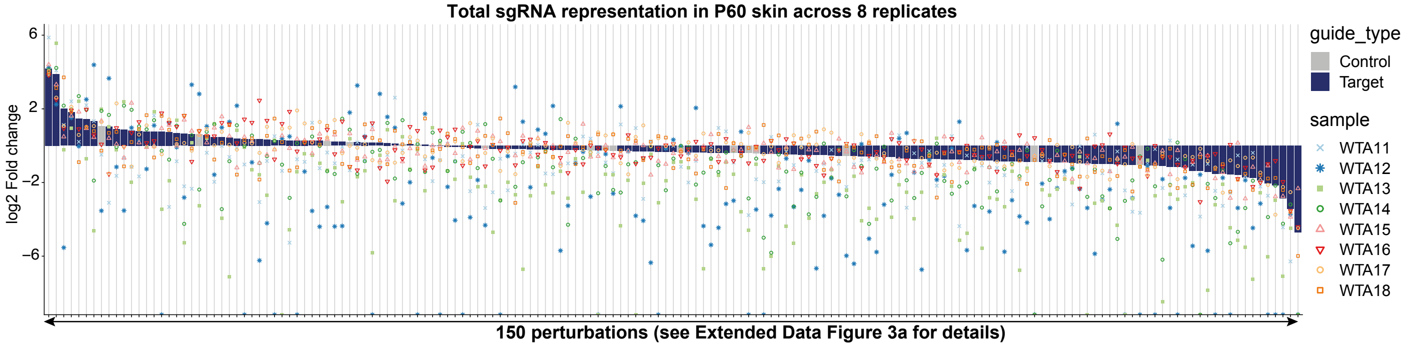

Supplementary Figure 2. Enrichment and depletion of the cancer gene perturbations in P60 skin across 8 replicates.
The enrichment and depletion patterns of total sgRNAs across the 8 replicates in the mouse P60 skin. The waterfall plot displays the enrichment and depletion of 150 perturbations at P60, represented as the log2 fold change between total cell numbers at P60 compared to the respective library T0, shown for all 8 replicates. The specific perturbation details can be found in Extended Data Figure 3a.

Supplementary Figure 3

a

TNF-α module (complete 25 genes)

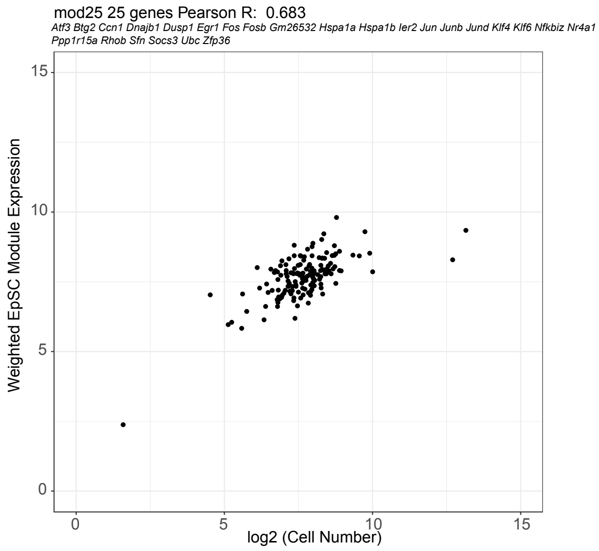

TNF-α module (17 genes implicated in TNF signaling)

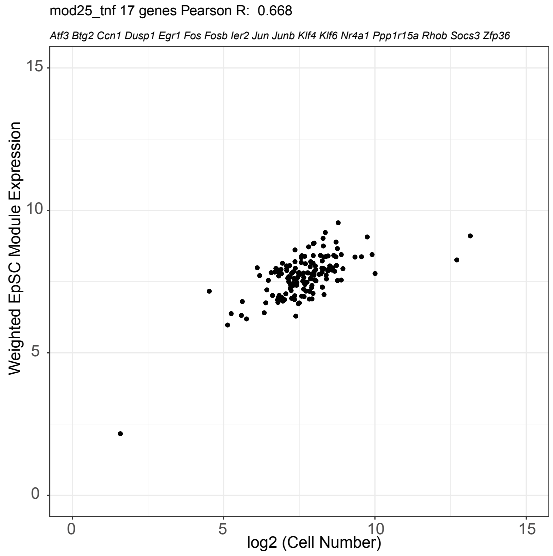

Extended Data Figure 4d, module gene GO terms

UV response

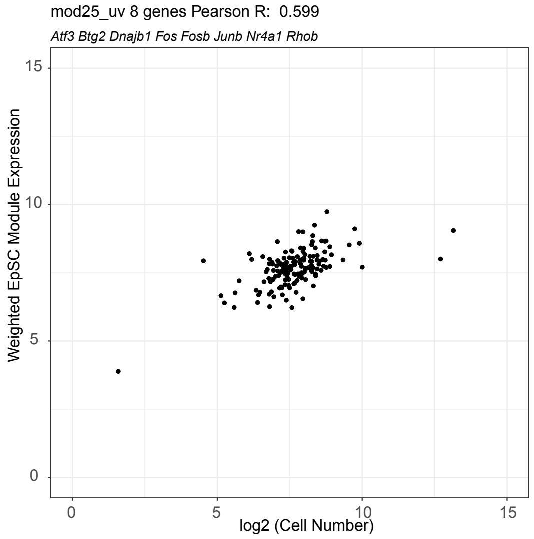

Hypoxia

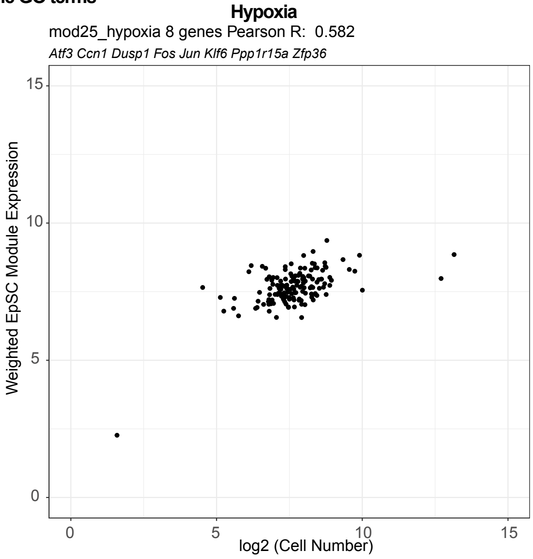

b

Figure 2m, Top 5 vs. middle 5 comparison

p53

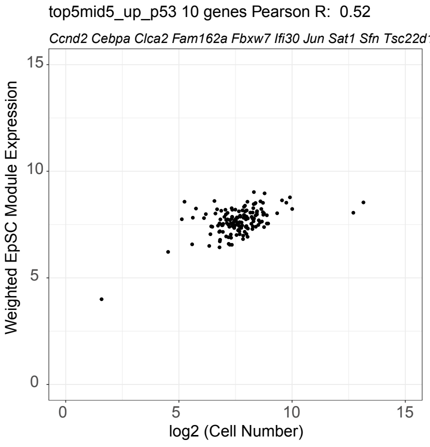

Apoptosis

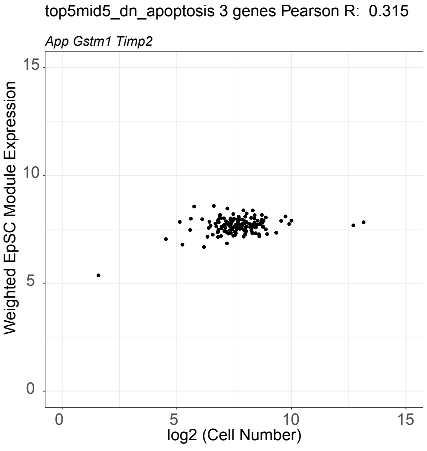

IL-2/STAT5 signaling

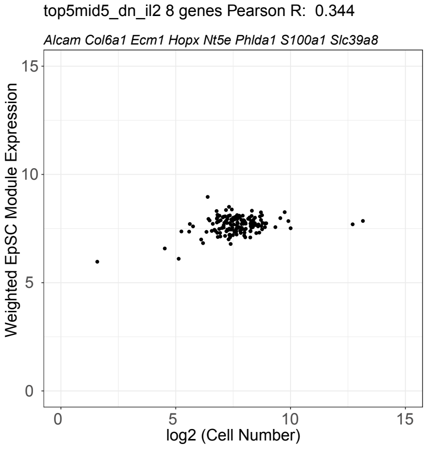

Supplementary Figure 3. The TNF-α signaling module explains a substantial portion of the variance in clonal expansion rates.
a, The model utilizes the average expression of 25 module genes, 17 module genes, UV response genes or hypoxia signaling genes in epidermal stem cells (gene sets in Extended Data Figure 4d). Pearson correlation of the fitted linear model is 0.683 for the 25 module genes, which explains a statistically significant proportion of variance ($p < 0.001$).
b, In contrast, the alternate gene cohorts from Figure 2m show lower Pearson correlation coefficients, indicating lower predictive power for clonal expansion rates relative to the TNF-α module.

Supplementary Figure 4

a

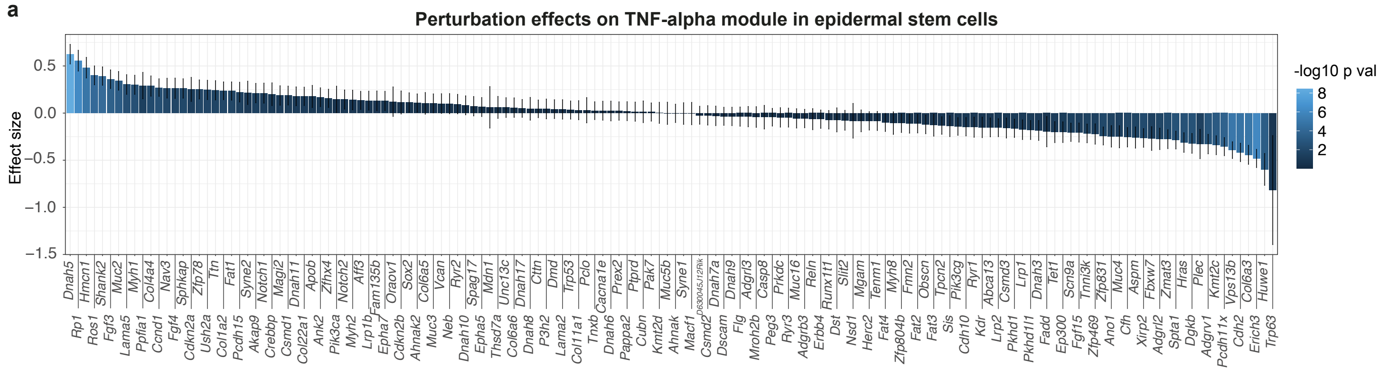

Supplementary Figure 4. Perturbation scores on TNF-α module in epidermal stem cells.
a, Effect size of each perturbation on the TNF-α module in epidermal stem cells compared to control sgRNAs reveals that *Dnah5* and *Rp1* perturbations result in the largest TNF-α module effect size. Error bars represent standard error of the linear regression. The p-values from the linear model were computed using a Wald t-distribution approximation..

Supplementary Figure 5

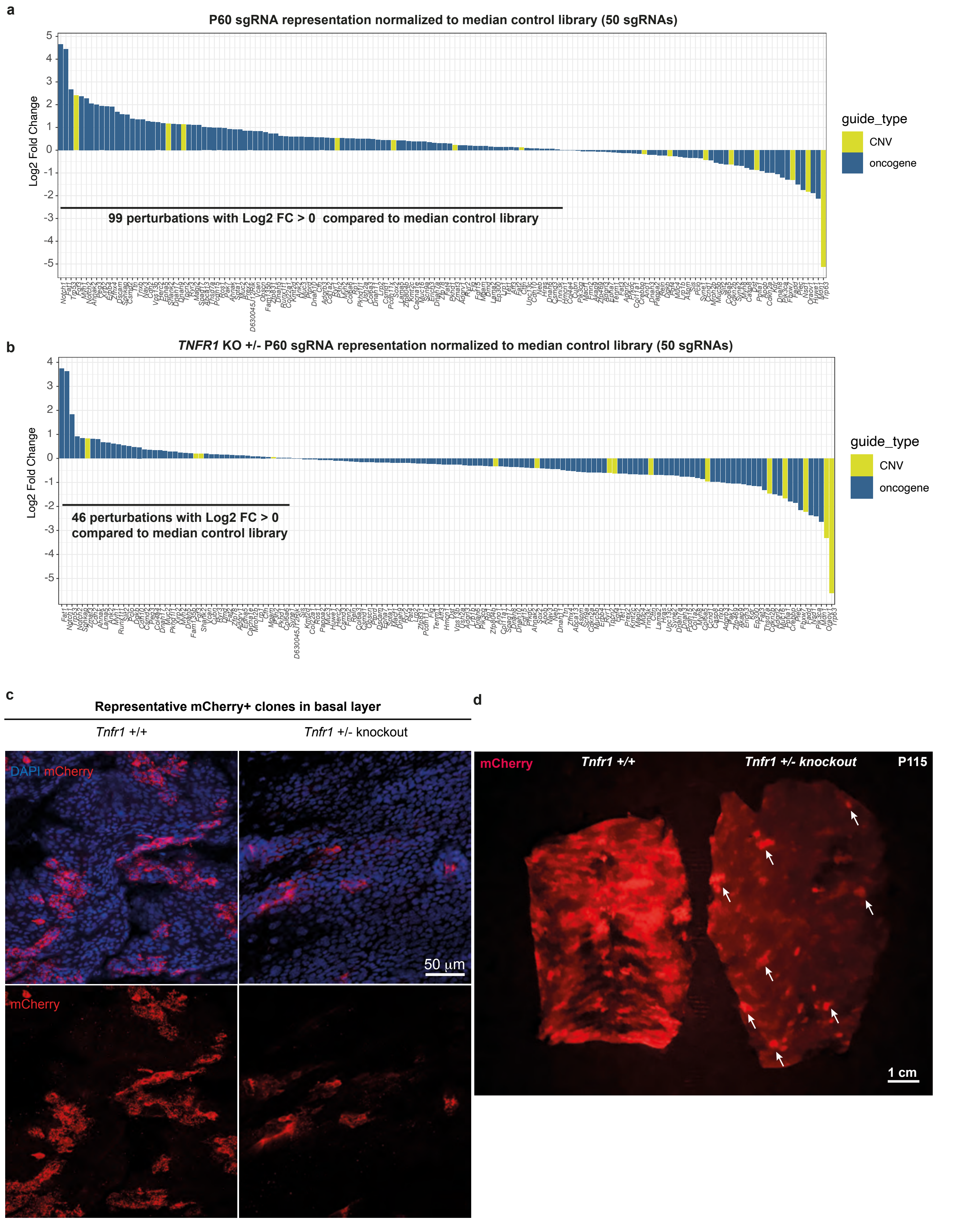

Supplementary Figure 5. The enrichment of cancer gene perturbations is dependent on TNFR1 (corresponding to Figure 3d).
a-b, Enrichment and depletion of the 150 cancer gene perturbations in wild-type and *Tnfr1 +/-* mutant skin. The library with 500 sgRNAs was microinjected into E9.5 embryos and the representation of this library was analyzed in P60 wild-type (a) and *Tnfr1* +/- (b) animals through amplicon sequencing. Using the MAGeCK algorithm, each perturbation was normalized to the median of the 50 control sgRNAs. While 99 perturbations show a log2 fold change >0 in wild-type P60 animals, only 46 perturbations exceeded this threshold in *Tnfr1 +/-* P60 animals, suggesting strongly reduced clone sizes. Data represent the average of 8 wild-type and 4 *Tnfr1* +/- replicates. CNV, copy number variations.
c, Representative mCherry areas in wild-type and *Tnfr1* +/- knockout P60 skin by wholemount stainings.
d, Differences in distribution of mCherry-positive perturbations in P115 wild-type compared to *Tnfr1 +/-* knockout skin suggest smaller mCherry-pos. areas in *Tnfr1 +/-* knockout skin (arrows). The library with 500 sgRNAs was injected at E9.5 into wild-type and *Tnfr1 +/-* knockout animals (same virus/day injection, parallel experimental arm). The complete backskin of the animals were collected and imaged at P115 (not P60).

Supplementary Figure 6

a **Genes with high skin SCC mutation rates are enriched in control vs. *Tnfr1* knockout p60 (project GENIE, corresponding to bottom panel in b)**

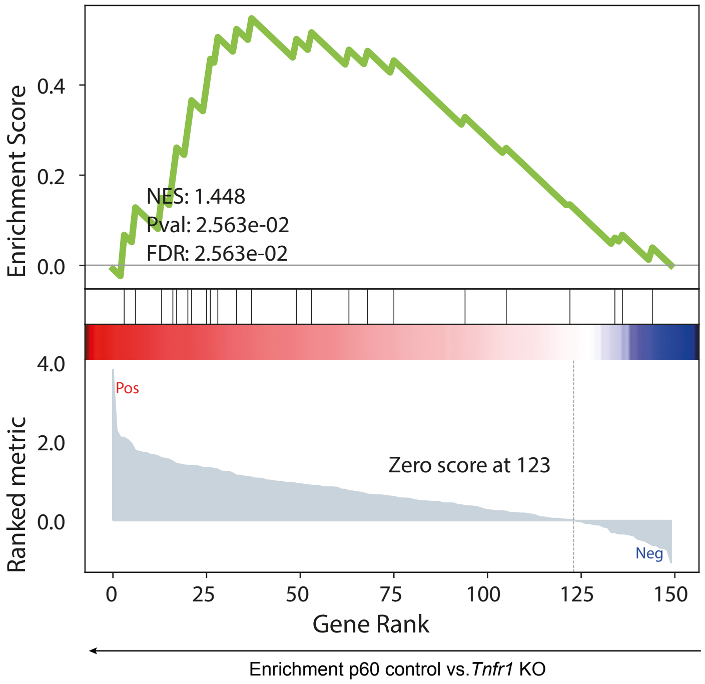

b

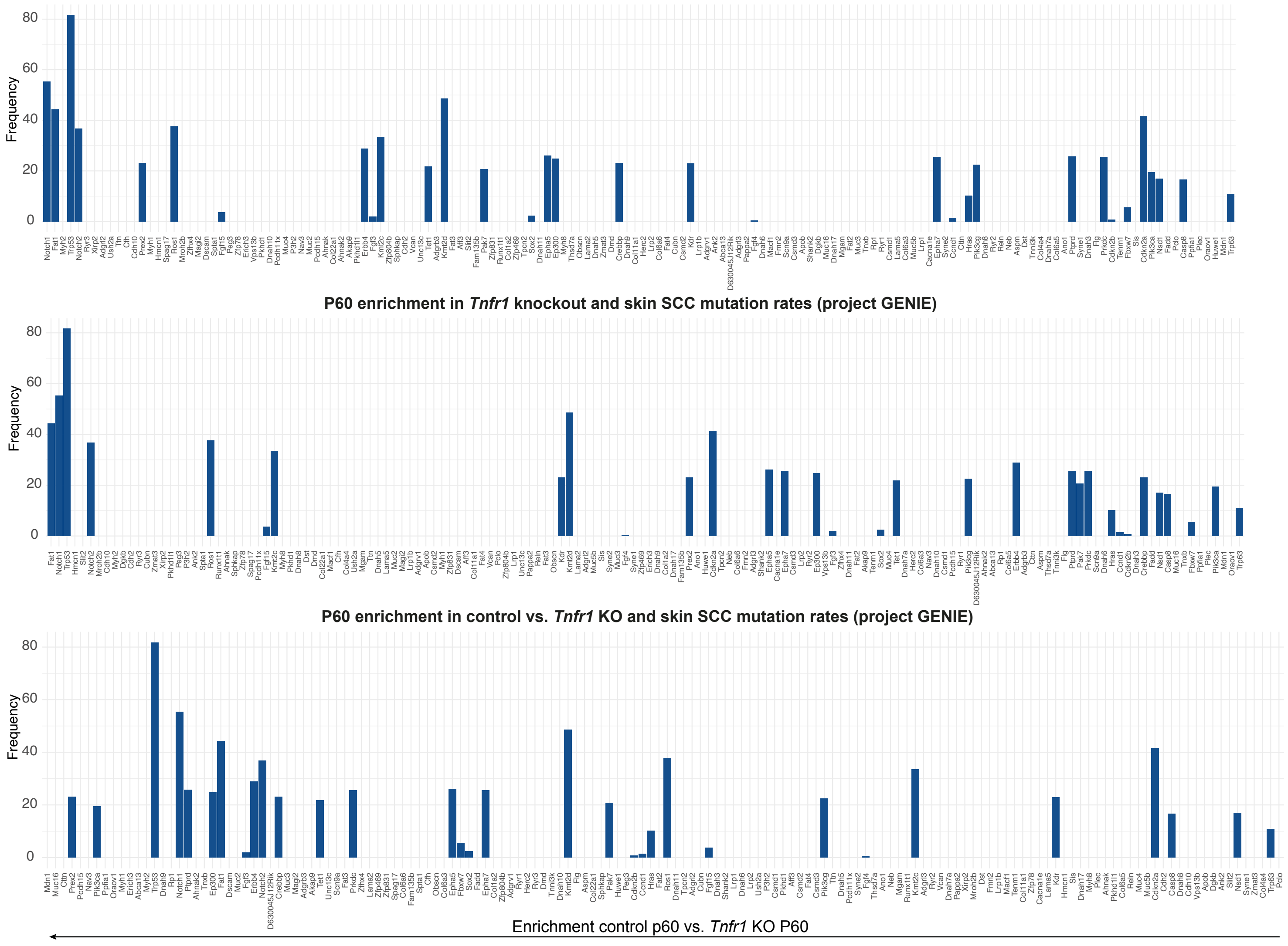

Supplementary Figure 6. Perturbations targeting genes with high mutation rates in skin SCCs are more highly enriched in control vs. *Tnfr1* mutant P60 skin.
a, Gene Set Enrichment Analysis (GSEA) reveals significant enrichment of genes with mutation rates > 15% in human skin squamous cell carcinomas (SCCs) in control compared to *Tnfr1* +/- mutant skin. These genes with mutation rates > 15% in skin SCCs, identified from the project GENIE (323 SCC samples), were used as a proxy for a gene's potential to drive aggressive cancer phenotypes. The analysis suggests that *Tnfr1* mutant skin show a significantly (FDR=0.0256, NES 1.448) reduced enrichment of aggressive perturbations. NES, Normalized Enrichment Score.
b, Representation of the skin SCC genes with mutation rates > 15% in P60 control (upper panel), *Tnfr1* mutant (middle panel) or control/*Tnfr1* mutant (bottom panel) P60 skin. The bottom panel corresponds to the GSEA in a. Empty boxes indicate genes not included in the project GENIE.

Supplementary Figure 7

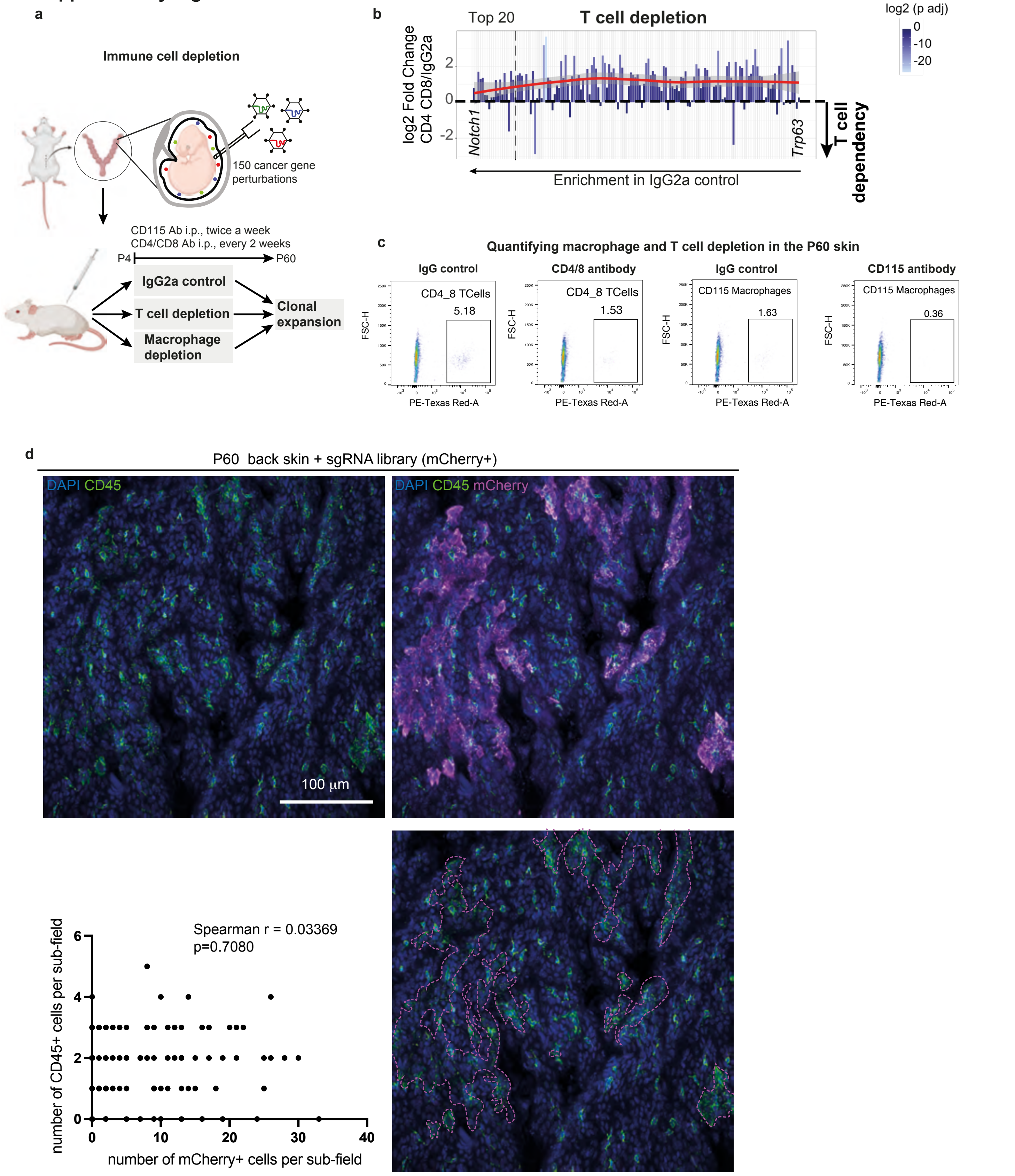

Supplementary Figure 7. T cell depletion does not reduce clonal expansion of cancer genes.

a, Schematic outline of the experimental strategy to deplete immune cells. The library of 500 sgRNAs was microinjected into E9.5 embryos. Between P4 and P60, T cells and macrophages were depleted by intraperitoneal (i.p.) injection of anti-CD4/CD8 and anti-CD115 antibodies (methods). IgG2a was injected i.p. as a control. At P60, epidermal cells were isolated and sgRNAs were amplified and sequenced.

b, T cell depletion did not result in a reduction in clonal expansions. Overall, T cell depletion rather induced a trend towards larger clonal expansions over all perturbations. See also Extended Data Table 5 for the results. CD4/8, 12 amplicon sequencing samples from 6 animals (anterior/posterior back skin).

c, Immune cell depletion protocol effectively depletes T cells and macrophages in the mouse skin. Percentages of T cells and macrophages in the P60 skin as determined by flow cytometry.

d, CD45-positive immune cells are not enriched in mCherry-positive skin regions. Number of CD45-positive and mCherry-positive cells were quantified per sub-field. There is no significant correlation in the number of CD45-positive and mCherry-positive cells.

Supplementary Figure 8

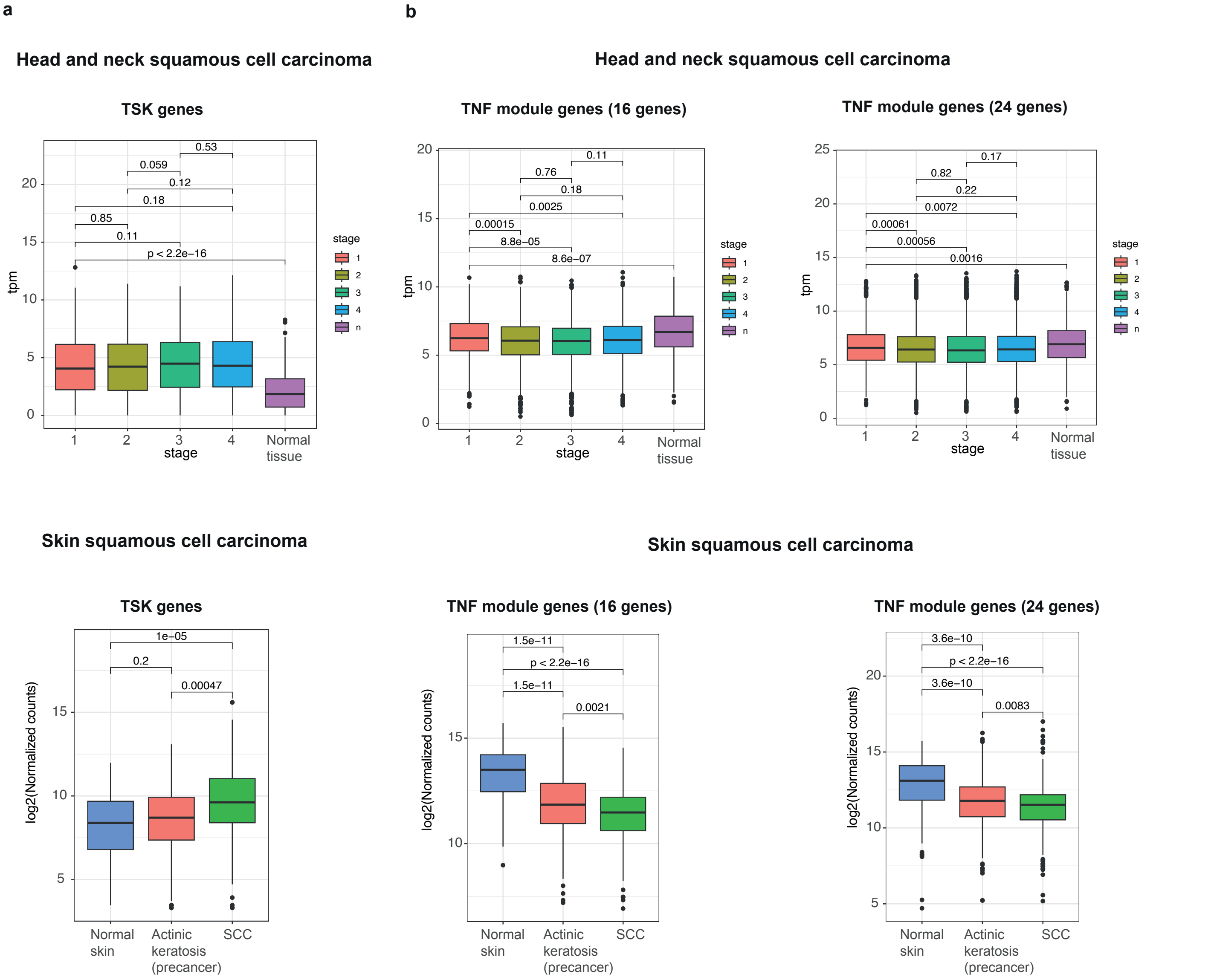

Supplementary Figure 8. TNF-α module downregulation and TSK signature upregulation in human SCC patients.
a, TSK gene expression is increased in human skin and head and neck SCC patients compared to normal tissue. Gene expression in head and neck SCC was extracted from TCGA (using Vivian et al., Nature Biotechnology, 2017, https://doi.org/10.1038/nbt.3772). Gene expression in actinic keratosis and skin SCC was extracted from Chitsazzadeh et al., Nature Communications, 2016, https://doi.org/10.1038/ncomms12601, GSE84293) as DESeq2 normalized counts.
Box plots indicate the interquartile range with median drawn as line and Tukey-style whiskers. Sample size shown in the plot title. P values indicate a two-tailed t-test.
b, TNF-α module gene expression is decreased in human skin and head and neck SCC patients compared to normal tissue. The decrease correlates with advanced disease stages in head and neck SCC patients. Gene expression in head and neck SCC was extracted from the TCGA (using Vivian et al., Nature Biotechnology, 2017, https://doi.org/10.1038/nbt.3772). Gene expression in actinic keratosis and skin SCC was extracted from Chitsazzadeh et al., Nature Communications, 2016, https://doi.org/10.1038/ncomms12601, GSE84293) as DESeq2 normalized counts.
Box plots indicate the interquartile range with median drawn as line and Tukey-style whiskers. Sample size shown in the plot title. P values indicate a two-tailed t-test.

Supplementary Figure 9

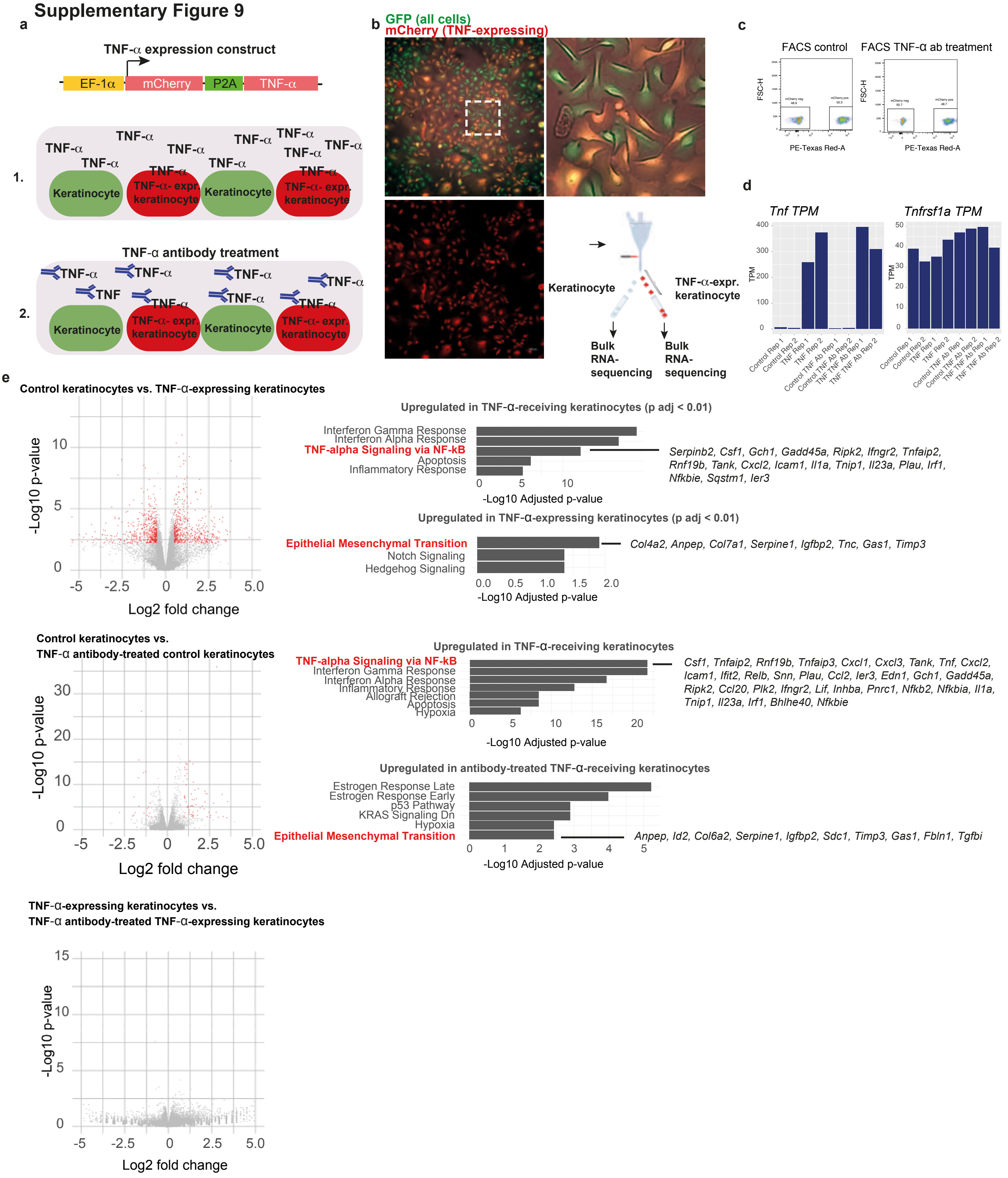

Supplementary Figure 9. The distinct role of the autocrine TNF-α gene program.
a-e, We conducted transcriptional analysis of co-cultured TNF-α-expressing and control keratinocytes. Both cell types were equally exposed to TNF-α (a), being mixed 1:1 on the same plate (b) and thus subjected to identical TNF-α levels in the media (secreted by the TNF-α-expressing cells). In a parallel experiment, the cells were treated with an anti-TNF-α antibody. TNF-α-expressing and control keratinocytes were then sorted and subsequently subjected to bulk RNA sequencing (c). Notably, despite this equal TNF-α exposure and similar levels of TNF receptor 1 (d), the two cell types displayed distinct transcriptional profiles. We identified 498 differentially expressed genes (adjusted p-value < 0.05, log2 FC >1 or < -1, DESeq2), highlighting the significant impact of the autocrine TNF-α gene program on keratinocyte gene expression, independent of the interaction between TNF-α and TNF receptor 1.
Cells receiving TNF-α showed an upregulation of genes associated with canonical TNF-α signaling pathways. Conversely, TNF-α-expressing cells demonstrated an upregulation of genes linked to epithelial-mesenchymal transition (EMT), aligning with the invasive characteristics promoted by the autocrine TNF-α gene program.
The comparisons also revealed that while TNF-α signaling can be inhibited by TNF-α antibody treatment in TNF-α-receiving cells (e), such treatment did practically not elicit any changes (only 2 diff. expressed genes with p adj < 0.05) in the gene expression of TNF-α-expressing cells. This suggests that TNF-α antibody treatment is ineffective in inhibiting the autocrine TNF-α gene program. Consequently, these observations suggest that TNF-α antibody therapies in cancer patients require a more nuanced understanding of their inhibitory effect on conventional versus autocrine TNF-α signaling.
Volcano plots, grey dots not significant, red dots p adj < 0.05 and log2 fold change > 1 or < -1.

Supplementary Figure 10

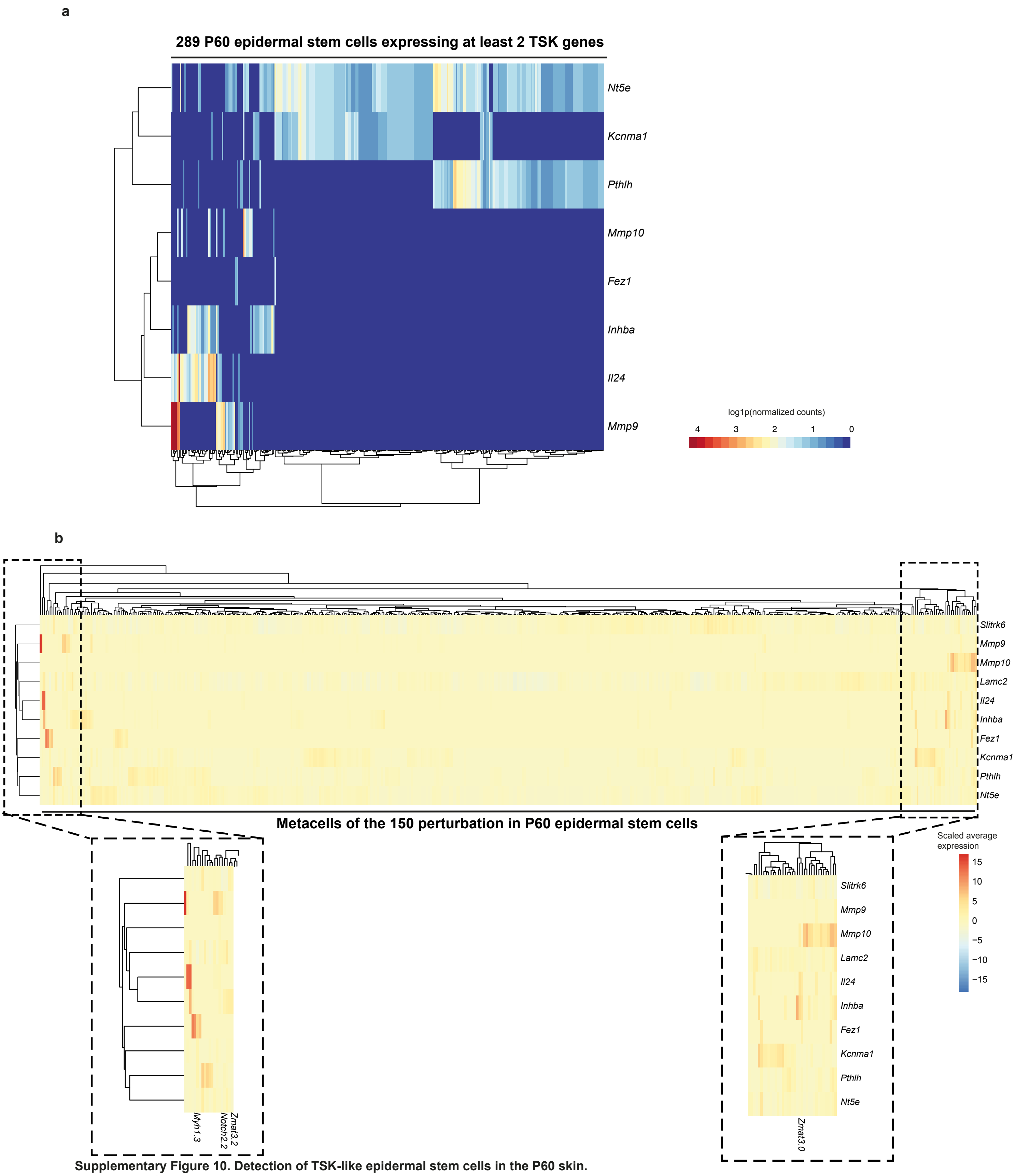

Supplementary Figure 10. Detection of TSK-like epidermal stem cells in the P60 skin.
a, Beside the SEACells analysis (Figure 5d, Extended Data Figure 8), we also assessed whether any perturbed P60 epidermal stem cells expressed sets of TSK genes. The analysis revealed 289 P60 epidermal stem cells expressing at least 2 TSK genes.
log1p(normalized counts) indicate log with 1 pseudocount.
b, SEACells identfies rare cell states (metacells) expressing TSK markers even in the P60 epidermal stem cell population. Heatmap shows average expression of the TSK marker genes. Boxed regions are magnified below and highlight metacells and their expression of TSK marker genes. *Zmat3* is the top selected perturbation between P60 skin and tumors (Figure 5c).

Supplementary Figure 11

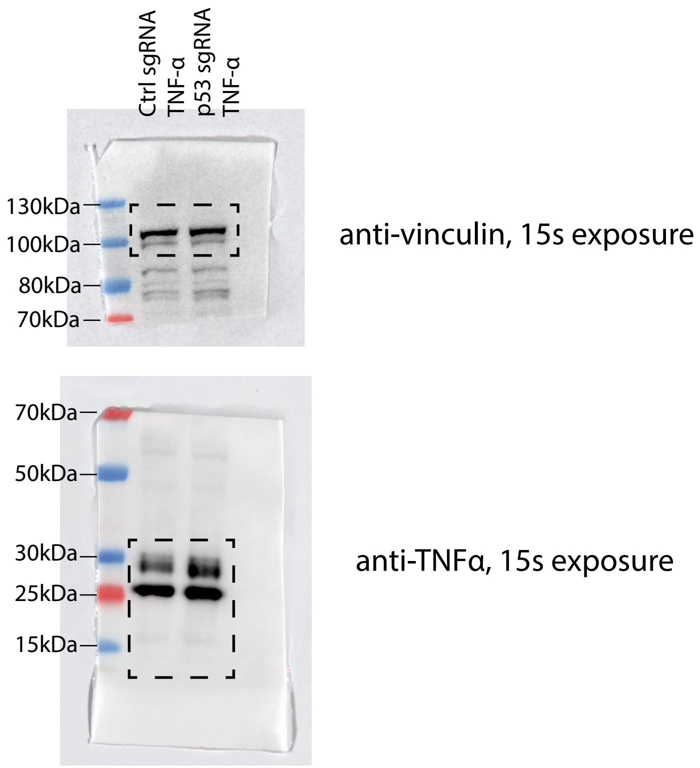

Supplementary Figure 11. Uncropped western blot images.
Lysate from lentiviral infected keratinocytes was run on the same gel. Membrane was cut after transfer for separate antibody incubation. Dashed boxes indicate the region shown in Figure 5f.
