# Supplementary material for: In vivo single-cell CRISPR uncovers distinct TNF programmes in tumour evolution
Source: Nature. 2024 Jul 17;632(8024):419–28. doi: 10.1038/s41586-024-07663-y (PMC11306103; doi:10.1038/s41586-024-07663-y)
Supplement: Supplementary file 2 — Reporting Summary [file 41586_2024_7663_MOESM2_ESM.pdf]

nature portfolio

Corresponding author(s): Ataman Sendoel, Andreas Moor

Last updated by author(s): Jul 4, 2024

Reporting Summary

Nature Portfolio wishes to improve the reproducibility of the work that we publish. This form provides structure for consistency and transparency in reporting. For further information on Nature Portfolio policies, see our Editorial Policies and the Editorial Policy Checklist.

Statistics

For all statistical analyses, confirm that the following items are present in the figure legend, table legend, main text, or Methods section.

n/a	Confirmed	
☐	☒	The exact sample size (*n*) for each experimental group/condition, given as a discrete number and unit of measurement
☐	☒	A statement on whether measurements were taken from distinct samples or whether the same sample was measured repeatedly
☐	☒	The statistical test(s) used AND whether they are one- or two-sided<br>*Only common tests should be described solely by name; describe more complex techniques in the Methods section.*
☐	☒	A description of all covariates tested
☒	☐	A description of any assumptions or corrections, such as tests of normality and adjustment for multiple comparisons
☐	☒	A full description of the statistical parameters including central tendency (e.g. means) or other basic estimates (e.g. regression coefficient) AND variation (e.g. standard deviation) or associated estimates of uncertainty (e.g. confidence intervals)
☐	☒	For null hypothesis testing, the test statistic (e.g. *F*, *t*, *r*) with confidence intervals, effect sizes, degrees of freedom and *P* value noted<br>*Give P values as exact values whenever suitable.*
☒	☐	For Bayesian analysis, information on the choice of priors and Markov chain Monte Carlo settings
☒	☐	For hierarchical and complex designs, identification of the appropriate level for tests and full reporting of outcomes
☐	☒	Estimates of effect sizes (e.g. Cohen's *d*, Pearson's *r*), indicating how they were calculated

Our web collection on statistics for biologists contains articles on many of the points above.

Software and code

Policy information about availability of computer code

Data collection	-Microscopy images were obtained using a Zeiss Axio Observer with Zen software (version 3.1)<br>-Western blot luminesence was recorded using the GE ImageQuant LAS4000 (version 1.2)<br>-Single cell capture was performed with the BD Rhapsody Scan and Analysis software (version 1.3.0.13)<br>-ELISA readings were taken using a Tecan i-control (version 2.0)<br>-Quant Studio Real-Time PCR software (version 1.3) was used to record qPCR results.<br>-Flow cytometry data was acquired with the FACSDiva software (BD Biosciences, version 8.0.1)
Data analysis	Single-cell RNA sequencing data were processed with the following tools/packages using the commands described in the methods section:<br>BD Rhapsody Pipeline hosted at the Seven Bridge's cloud platform (https://www.sevenbridges.com/, version 1.8-1.9.1)<br>STAR aligner (version 2.7.10b)<br>Scrublet Python Package (version 0.2.3)<br>MAST package (version 1.26.0)<br>pheatmap package (version 1.0.12)<br>pyMDE package (version 0.1.15)<br>MAGECK package (version 0.5.9)<br>WGCNA package (version 1.72-1)<br>metap package (version 1.8)<br>Cellchat (version 1.6.0)<br>RPCA (version 0.2.3)<br>SEACells (version 0.3.3)<br>EnrichR (version 3.2)

GSEApy (version 1.0.5)
DeSeq2 (version 3.18)
ImageJ (stable release 1.54h)

Spatial transcriptomics data:
Space Ranger software (version 2.0.1, 10x Genomics)
spacexr package (version 2.2.1, https://github.com/dmcable/spacexr)
Seurat R package (version 4.0.3)
ISCHIA package (version 1.0.0.0)

qPCR data was analysed with the Quant Studio Real-Time PCR software (version 1.3) and Microsoft Excel 365(Version 2302)
Immunofluoresence pictures were taken with the ZEN Blue microscopy software (version 3.1). Images shown are maximum intensity projections.
Flow cytometry data were analyzed both with the BD FACSDiva (BD Biosciences, version 8.0.1) and FlowJo (version 10.8.1).

Custom scripts developed in this study is available at https://github.com/sendoellab/single-cell_CRISPR

For manuscripts utilizing custom algorithms or software that are central to the research but not yet described in published literature, software must be made available to editors and reviewers. We strongly encourage code deposition in a community repository (e.g. GitHub). See the Nature Portfolio guidelines for submitting code & software for further information.

Data

Policy information about availability of data

All manuscripts must include a data availability statement. This statement should provide the following information, where applicable:

- Accession codes, unique identifiers, or web links for publicly available datasets
- A description of any restrictions on data availability
- For clinical datasets or third party data, please ensure that the statement adheres to our policy

The complete single-cell RNA sequencing and CROP-seq data for P4 stage, P60 stage, tumor amplicon sequencing and RNA-sequencing data are available on the GEO GSE235325.
Gene expression data in actinic keratosis and skin SCC was extracted from Chitsazzadeh et al., Nature Communications, 2016, https://doi.org/10.1038/ncomms12601.
Molecular signature database hallmark genes for gene set enrichment analysis was accessed via https://www.gsea-msigdb.org/gsea/msigdb/mouse/collections.jsp
TCGA data was accessed via xena.ucsc.edu.

Research involving human participants, their data, or biological material

Policy information about studies with human participants or human data. See also policy information about sex, gender (identity/presentation), and sexual orientation and race, ethnicity and racism.

Reporting on sex and gender	not applicable
Reporting on race, ethnicity, or other socially relevant groupings	not applicable
Population characteristics	not applicable
Recruitment	not applicable
Ethics oversight	not applicable

Note that full information on the approval of the study protocol must also be provided in the manuscript.

Field-specific reporting

Please select the one below that is the best fit for your research. If you are not sure, read the appropriate sections before making your selection.

☒ Life sciences ☐ Behavioural & social sciences ☐ Ecological, evolutionary & environmental sciences

For a reference copy of the document with all sections, see nature.com/documents/nr-reporting-summary-flat.pdf

Life sciences study design

All studies must disclose on these points even when the disclosure is negative.

Sample size	Sample size was not predetermined by statistical methods. Based on previous screens and general coverage requirements for high-content CRISPR screenings (Bock, C., Datlinger, P., Chardon, F. et al. High-content CRISPR screening. Nat Rev Methods Primers 2, 8 (2022). https://doi.org/10.1038/s43586-021-00093-4; Xin Jin et al. In vivo Perturb-Seq reveals neuronal and glial abnormalities associated with autism risk

	genes. Science 370, eaaz6063 (2020). DOI:10.1126/science.aaz6063), we aimed for a coverage of our sgRNA library of at least 200x (cells/sgRNA) in the in vivo samples. We achieved a coverage of 240x for the P4 and 366x for the P60 skin.
Data exclusions	For single cell CRISPR analysis, low quality and doublet cells were filtered out following standard filtering parameters. Details are provided in the methods section.
Replication	For single-cell RNA sequencing data, we performed 7 replicates for the P4 time point, resulting in a total number of 120,077 cells after filtering and sgRNA annotation. For the P60 time point, we performed 8 replicates, resulting in a total number of 183,084 cells after filtering and sgRNA annotation.<br>For TNFR1-dependency (Fig. 3c), 8 wild-type and 4 Tnfr1 replicates were performed.<br>Tumor single-cell data were derived from 30 individual tumors from 2 animals, separated into mCherry-positive and mCherry-negative cells. scRNA-seq library preparations were performed separately for mCherry-positive and mCherry-negative cells.<br>ELISA was carried out in biological and technical triplicates.<br>MTT assay was carried out in 5 biological replicates and technical triplicates.<br>in vivo EdU assay was quantified across 12 separate sections with areas of 25 cells or more<br>qPCR was carried out in biological triplicates and technical duplicates.<br>All attempts at replication were successful.
Randomization	For mouse experiments, mice of relevant genotype were randomly assigned to each experimental protocol.
Blinding	Investigators were not blinded during data acquisition. Since most of our readouts are sequencing-based, blinding would not be expected to have an effect on the experimental outcome.

Reporting for specific materials, systems and methods

We require information from authors about some types of materials, experimental systems and methods used in many studies. Here, indicate whether each material, system or method listed is relevant to your study. If you are not sure if a list item applies to your research, read the appropriate section before selecting a response.

Materials & experimental systems

n/a	Involved in the study
☐	☒ Antibodies
☐	☒ Eukaryotic cell lines
☒	☐ Palaeontology and archaeology
☐	☒ Animals and other organisms
☒	☐ Clinical data
☒	☐ Dual use research of concern
☒	☐ Plants

Methods

n/a	Involved in the study
☒	☐ ChIP-seq
☐	☒ Flow cytometry
☒	☐ MRI-based neuroimaging

Antibodies

Antibodies used	Primary antibodies for immunofluoresence:<br>rat anti-RFP (Chromotek, 5F8; 1:200), rabbit anti-RFP (MBL, PM005; 1:200), chicken anti-GFP (Abcam, ab13970; 1:200), rat anti-CD45-biotin (Biolegend, 103104; 1:200), goat anti-TNFR1 (R&D, AF-425-PB; 1:200), rabbit anti mouse TNF-alpha (CST #11948 [D2D4] XP[R, 1:300), rat Anti-Mouse CD104 aka ITGB beta 4 (BD Pharmingen 553745, 1:300), chicken anti-Keratin14 (Biolegend 906001, 1:300).<br>Secondary antibodies for immunofluoresence:<br>All secondary antibodies used were raised in a donkey host and were conjugated to Alexafluor488(Codes: 703-545-155, 712-545-150, 712-545-153), Cy3(Codes:703-165-155, 711-165-152), or AlexaFluor647(Codes:703-605-155,711-605-152,712-605-153) (Jackson ImmunoResearch Laboratory; 1:500- 1:1000).<br><br>Primary antibodies for western blot:<br>Anti-TNF-α (Cell Signaling Technology, #11948), Anti-Vinculin (Abcam, ab129002)<br>Secondary Antibodies Western Blot:<br>Goat Anti-Rabbit IgG HRP Linked Antibody (Cell Signaling Technology 7074S)<br><br>Antibodies for Immune Cell depletion:<br>rat anti-mouse CD4 (YTS 191.1, Hoelzel Diagnostika LEIN-C3210)<br>rat anti-mouse CD8 (YTS169.4 Hoelzel Diagnostika LEIN-C2850)<br>rat anti-Mouse CD115 (CSF-1R, Hoelzel Diagnostika LEIN-C2268)<br>rat IgG isotype control (Hoelzel Diagnostika, LEIN-I-1177).
Validation	Antibodies were validated by the manufacturer and multiple publications. Anti-TNF-α antibody was additionally validated by western blot and immunofluorescence in TNF-α-negative cells.<br><br>rat anti-RFP (Chromotek, 5F8) - Zhang Y, Rózsa M, Liang Y, et al. Fast and sensitive GCaMP calcium indicators for imaging neural populations. Nature. 2023;615(7954):884-891. doi:10.1038/s41586-023-05828-9

rabbit anti-RFP (MBL, PM005) - Ishida Y et al. Autophagic elimination of misfolded procollagen aggregates in the endoplasmic reticulum as a means of cell protection. Mol Biol Cell. 20, 2744-54 (2009)(PMID:19357194)

chicken anti-GFP (Abcam, ab13970) - Berg EM et al. Brainstem circuits encoding start, speed, and duration of swimming in adult zebrafish. Neuron 111:372-386.e4 (2023)

rat anti-CD45-biotin (Biolegend, 103104) - Podd BS, Thoits J, Whitley N, et al. T cells in cryptopatch aggregates share TCR gamma variable region junctional sequences with gamma delta T cells in the small intestinal epithelium of mice. J Immunol. 2006;176(11):6532-6542. doi:10.4049/jimmunol.176.11.6532

goat anti-TNFR1 (R&D, AF-425-PB) - Knizkova D, Pribikova M, Draberova H, et al. CMTM4 is a subunit of the IL-17 receptor and mediates autoimmune pathology. Nat Immunol. 2022;23(11):1644-1652. doi:10.1038/s41590-022-01325-9

rabbit anti mouse TNF-alpha (CST #11948) - Chan L, Chung CC, Yu RC, Hong CT. Cytokine profiles of plasma extracellular vesicles as progression biomarkers in Parkinson's disease. Aging (Albany NY). 2023;15(5):1603-1614. doi:10.18632/aging.204575

rat Anti-Mouse CD104 aka ITGB beta 4 (BD Pharmingen 553745) - Giancotti FG. Signal transduction by the alpha 6 beta 4 integrin: charting the path between laminin binding and nuclear events. J Cell Sci. 1996;109 (Pt 6):1165-1172. doi:10.1242/jcs.109.6.1165

chicken anti-Keratin14 (Biolegend 906001) - Radtke AJ, Kandov E, Lowekamp B, et al. IBEX: A versatile multiplex optical imaging approach for deep phenotyping and spatial analysis of cells in complex tissues. Proc Natl Acad Sci U S A. 2020;117(52):33455-33465. doi:10.1073/pnas.2018488117

Anti-Vinculin (Abcam, ab129002) - Hühn D, Martí-Rodrigo P, Mouron S, et al. Prolonged estrogen deprivation triggers a broad immunosuppressive phenotype in breast cancer cells. Mol Oncol. 2022;16(1):148-165. doi:10.1002/1878-0261.13083

Goat Anti-Rabbit IgG HRP Linked Antibody (Cell Signaling Technology 7074S) - Sakamoto A, Inoue H, Miyamoto S, Ito S, Soda Y, Tani K. Coxsackievirus A11 is an immunostimulatory oncolytic virus that induces complete tumor regression in a human non-small cell lung cancer. Sci Rep. 2023;13(1):5924. Published 2023 Apr 12. doi:10.1038/s41598-023-33126-x

Jackson Immuno Goat secondary for IF - Ha J, Shin J, Seok E, Kim S, Sun S, Yang H. Estradiol and progesterone regulate NUCB2/nesfatin-1 expression and function in GH3 pituitary cells and THESC endometrial cells. Anim Cells Syst (Seoul). 2023;27(1):129-137. Published 2023 Jun 20. doi:10.1080/19768354.2023.2226735

rat anti-mouse CD4 (YTS 191.1, Hoelzel Diagnostika LEIN-C3210) - 1. Tallón de Lara, P. et al. CD39+PD-1+CD8+ T cells mediate metastatic dormancy in breast cancer. Nat. Commun. 2021 121 12, 1–14 (2021).
rat anti-mouse CD8 (YTS169.4 Hoelzel Diagnostika LEIN-C2850) - 1. Tallón de Lara, P. et al. CD39+PD-1+CD8+ T cells mediate metastatic dormancy in breast cancer. Nat. Commun. 2021 121 12, 1–14 (2021).
rat anti-Mouse CD115 (CSF-1R, Hoelzel Diagnostika LEIN-C2268) - 1. Lelios, I. et al. Monocytes promote UV-induced epidermal carcinogenesis. Eur. J. Immunol. 51, 1799 (2021).

Eukaryotic cell lines

Policy information about cell lines and Sex and Gender in Research

Cell line source(s)	Lenti-X(TM) 293T cells for virus production were purchased from TaKaRa Clontech (632180).<br>Mouse keratinocyte cells were derived in-house as described in the method section.
Authentication	Commercially available cell lines were authenticated by the vendor. No additional authentication was performed.
Mycoplasma contamination	Cell lines were tested for mycoplasma every three months using the Mycoplasma PCR detection kit and confirmed negative (Sigma; D9307).
Commonly misidentified lines<br>(See ICLAC register)	none of the misidentified lines were used in this study

Animals and other research organisms

Policy information about studies involving animals; ARRIVE guidelines recommended for reporting animal research, and Sex and Gender in Research

Laboratory animals	Tg(B6J.129(Cg)-Gt(ROSA)26Sortm1.1(CAG-cas9*,-EGFP)Fezh/J (denoted as “B6.Cas9”) were purchased from the Jackson Laboratory (strain #026179).<br>B6.129S Tnfrsf1atm1ImxTnfrsf1btm1Imx (denoted as Tnfr1-ko, originally from Jackson lab #003243) were acquired through the Swiss Immunology Mouse Repository (SwImMR). Wildtype mice of the strain CD1-IGS (denoted as “CD1”) were purchased from Charles River.<br>Animals were sacrificed as indicated in the manuscript for the single-cell data at either postnatal day 4(P4), or 60(P60). Immune depleted animals also at P60. DMBA/TPA treated animals were sacrificed at age postnatal day P144. TNF-overexpression animals (Fig. 5) were sacrificed at either P4, 17 or 27
Wild animals	No wild animals were used in this study.

Reporting on sex	P4 and P60 single-cell data contains both male and female animals.<br>TNFR1 dependency on P60 animals included both P60 male and female animals.<br>For the DMBA/TPA, chemical carcinogenesis tumor single-cell data, only female animals were used as recommended because male mice may fight, causing damage to the dorsal skin. (Filler, R. B., Roberts, S. J., & Girardi, M. (2007). Cutaneous two-stage chemical carcinogenesis. Cold Spring Harbor Protocols, 2007(9), pdb-prot4837.)
Field-collected samples	No field collected samples were used in this study.
Ethics oversight	The Animal Welfare Committee of the Canton of Zurich approved all animal protocols and experiments performed in this study (animal permits ZH074/2019, ZH196/2022)

Note that full information on the approval of the study protocol must also be provided in the manuscript.

Flow Cytometry

Plots

Confirm that:

☒ The axis labels state the marker and fluorochrome used (e.g. CD4-FITC).

☒ The axis scales are clearly visible. Include numbers along axes only for bottom left plot of group (a 'group' is an analysis of identical markers).

☒ All plots are contour plots with outliers or pseudocolor plots.

☒ A numerical value for number of cells or percentage (with statistics) is provided.

Methodology

Sample preparation	Single-cell suspension was achieved with mechanical dissociation and trypsin digest as described in the method section.
Instrument	BD FACSAria III
Software	BD FACSDiva (BD Biosciences, version 8.0.1) and FlowJo (version 10.8.1)
Cell population abundance	mCherry-positive rates were between 2.4%-16.3%. We sorted between 300,000-400,000 cells per replicate.
Gating strategy	1) FSC-A vs SSC-A was used to gate for the bulk population of cells<br>2) FSC-A vs FSC-H was used to minimize doublet sorting<br>3) DAPI-A vs FSC-H was used to gate for live cells<br>4) PE-Texas Red- A vs FSC-H was used to gate for lentivirus-infected cells<br>Please also see Extended Data Figure 1d, which exemplifies the gating strategy.

☒ Tick this box to confirm that a figure exemplifying the gating strategy is provided in the Supplementary Information.
